# The Nursing Supplement

**Published:** 1888-07-21

**Authors:** 


### The Nursing Mirror.

All Contributions for this Supplement should be addressed "The Nursing Mirror," care of The Editor, 38, Old Jewry, E.C.

### En Passant.

A COUNTRY CONVERSATION.—*Nurse:* "I fear, Mrs. Turnip, if you don't let baby undergo an operation, you will greatly regret it when he grows up!" *Mrs. Turnip:* "Well, Nurse, it shall be as you say; only he shan't go into one of them nasty hospitals." *Nurse:* "Why nasty, Mrs. Turnip? The operation cannot be properly done here." *Mrs. Turnip:* "Well, my child shall never go to a hospital, that's certain, that is! Don't I know well enough how they mix up the babies there? And small blame to 'em, say I. Babies is all alike as peas in a pod, but I ain't a-going to risk having some drunkard's child in return for my own. No, Nurse, tain't no use for you to say it ain't true; I've known it happen—of course it must where there are fifty babies together, all in beds alike. I daresay they'd give me back a boy again, and I'm sure I should never b'lieve t'were my own child. No, Nurse, he don't go to a hospital, never!"

NURSING THE INSANE.—An inspector of asylums writes to us concerning the necessary qualifications for attendants on the insane. He maintains that in all forms of nursing, as in all affairs of life, the higher the intelligence the better the execution of work. Besides the many good qualities which distinguish ordinary nurses, such as cleanliness, kindness, conscientiousness, &c., asylum attendants must possess robust health, courage, natural cheerfulness (not insipid amiability) and an excellent temper. Many are the trials of patience and endurance made on those who have charge of the insane, and in well-managed asylums they are trained to their difficult duties, and if they excel are promoted. Those having natural gifts, combined with a fair amount of intelligence, often succeed, where those of better social position and education fail. Keen observation alone can teach the words which soothe and the words which irritate the insane. A sentence an ordinary hospital nurse might speak, thinking it had no special point, is often misinterpreted by the insane, and excites them to anger. The good attendant knows the words which tranquillise the unhappy patient, and soothe the constant captiousness. Perhaps the conclusion of the whole matter lies in the fact that attendants, like poets, are born, not made.

THE SLUMS OF MANCHESTER.—There are now appearing in a Manchester paper some articles on nursing in the slums of that town. It seems there is good work being done there by the Ladies' Sanitary Association and the Sick Poor Nursing Institution, the second devoted to restoring health, the first to maintaining it. The writer says: "The value of skilled nursing in dangerous illnesses has until recently been quite unknown amongst the poorer classes. Hitherto the medicine bottle was their sheet anchor. With them, as long as the patients swallowed mixture after mixture, they were all right, and no other precautions were needed. Poulticing, regular feeding, washing, and keeping in a uniform temperature, as far as practicable, were looked upon as a waste of time and useless trouble. The making of beef tea, which they are taught to do by the nurses, was also totally unknown amongst them." Nor are the simplest laws of health ever heeded by most dwellers in crowded courts. There is much to be done in this way by ladies who will go from house to house and demonstrate the advantages of open windows and filtered water. To be a sanitary mission woman must necessarily be next to being a Bible mission woman, for cleanliness is next to godliness.

EALING COTTAGE HOSPITAL.—Under the heading of "The Nightingale Fund" we mentioned that the appointment of matron to the above hospital had been gained by a St. Thomas's nurse. Miss Reid writes to correct this statement, saying she has filled that situation ever since the hospital was establisbed in 1870. Two years ago the hospital was enlarged, when two nurses were appointed, one of whom, the head nurse, came from St. Thomas's Hospital. We apologise to Miss Reid for our mistake, and we also apologise to "The Nursing Record," for having lead it astray; for in copying our news, as usual, it has copied our mistake.

A WOMAN'S PARADISE.—Under the above heading Mrs. J. M. E. Saxby has been describing Western America, and once more advising labouring women to emigrate to the big towns in the great prairie territory. They are there promised high wages and chivalrous followers, and finally marriage and a good position in society at Ottawa. All this sounds very well, and Canada is the land of promise in many a nurse's dreams, but it is well to remember that very good nurses have failed in America. When the St. John's Community left Charing Cross Hospital, Sisters Amy and Anna took a few nurses over to Canada; but though they got much work they got little pay, and the nurses didn't like to be frozen in their beds, so they all returned to the old country. A lady writing from Ottawa to a friend who thought of taking up nursing out there says: "I know there are very few trained nurses about. There are four in the Convalescent Home, and they go out private nursing sometimes, and are very well paid, I have heard. If you come provided with good medical and other testimonials and a few introductions, the doctors here would be sure to help you. People from the old country are always warmly welcomed. But be sure you get introductions to either doctors or clergymen before you start, for it is an expensive journey to undertake as a speculation." Evidently the gist of the whole matter lies in having friends on the other side.

A BRAVE NURSE.—The authenticity of the news we gave under this heading having been called into question, we now publish the letter on which our information was based: "Dear Madam,—Nurse Finnis is quite well; she did not suck the tube, she blew through it. I must say she hardly left the dear child for two days and nearly two nights, and did all that she possibly could to prolong life.—Yours obediently, J. Yarwood, matron, Lt. William's Hospital. June 26th, 1888." We wish to express our great regret at the discourtesy we have quite unintentionally called down on the head of Miss Yarwood, who so kindly forwarded us information. At the same time, the sentiments of our contemporary, which doubted Miss Yarwood's word, are so evidently inspired merely by personal jealousy and dislike of ourselves, that we hope our kind correspondent will not heed them. If our contemporary is meeting with such extraordinary success as it states, surely this jealousy of our humble pages might decrease. Also it might venture to acknowledge whence it derives all the news it does us the honour to copy from our pages. Should any of our correspondents chance to notice the comments on their lack of education and grammar in this same contemporary, we venture to reassure them by pointing out that a journal whose first number contained such a sentence as "We breathe freer now" is scarcely to be taken as a judge of learning or correctness. So long as our correspondents send us such interesting letters as at present, we hope to print them as we receive them, and not to limit our letter-box to the reception of adulatory rhapsodies of our own articles. A mutual admiration society of six people may be interesting to its members, but not to the public.

### The Life of a Private Nurse.

For some time past I have made it my study and interest to find out how a private nurse's life is spent, and I must say that I think it a very lonely one. Perhaps some of my readers may say that we know already what their lives are, but I hope they will be patient with me, and hear my view, as I speak from experience.

Take a probationer who has been trained for private work. She has been brought up surrounded by good friends, and her relatives know that she has taken up a good and useful calling, yet they are often disappointed with her because she becomes absorbed in her work. Then, when she goes home for her annual holiday, she is changed; she is often no longer one of them: her ways are her own, if I may use the expression. Others, perhaps, are orphans; fate has not been so kind to them as to give them friends and kindred, and they become birds of passage. And there are many others that stand alone in this busy world of ours.

If they go for a holiday in the intervals of cases, they often go alone, and have to pay for it, which they often cannot afford. Never was there such a *corps de femmes* as the nursing corps in England before.

When a probationer or nurse takes up private work, she is often very young; I have known some only twenty-one or two years of age. At first the constant change of place and scene is new and fresh to her, and she likes it; the entire charge of a case is in her hands during the absence of the doctor for whom she is working, and this gives her a pleasant sense of importance. She is a happy woman, if the people around her are kind and considerate, though even then that does not make the responsibility lighter; but it makes it brighter, and the trials it involves easier to be borne. She has no one to confide in—to tell her troubles to, or to sympathize with her, or brighten her when she is low spirited. She seldom makes a lasting friendship, and thus her life is spent among strangers and in the solitude of the sick room. In a few years the novelty wears off and she is a young woman still. She seldom marries, because she moves in a small world of her own, and she seldom knows what goes on outside her nursing world. She is not allowed to go anywhere except in uniform, and in some institutions nurses are only allowed when in the home to go out for an hour or two daily, which is not only unkind but cruel. But that is the exception rather than the rule.

Are not nurses and the heads of institutions becoming fanatical in their devotion to duty? During an interval of cases the nurse is not allowed to go anywhere—to a theatre or a dance, for example—because it is un-nurse-like, &c.

In fact, she is seldom allowed to go to any place of amusement—she, who wants innocent amusement more than any one. How can she keep up that bright appearance so necessary to her if she does not have some relaxation? Everyone else has a little pleasure, why should not she? If a nurse is invited to go to a party, or dance, or anywhere else, and can give a satisfactory account of where she is going, why may she not go, and out of uniform if necessary. We are told that there is a time for all things: there is a time to dance and to make merry, as well as to walk on the stern path of duty. The rules of many institutions run thus: Nurses must be quiet in demeanour. No laughing, singing, &c., to be heard when in the Home." Is not this both foolish and cruel? I know that there must be discipline, but why should a number of young people be made like so many automatic pieces of machinery? They have not renounced the world and its pomps and vanities. Life was not given to us to be made a sacrifice in that way. All work and no play has the proverbial effect. What life is there so lonely as a private nurse's? yet she is expected to be a perfect woman. In hospital she has other young friends. In private work she is often quite alone. She is often months at a case, perhaps on night duty with a childish old lady or gentleman. After breakfast she goes for her daily walk, but often she feels too tired or too lonely to go far, and maybe she is in the country, miles away from anyone she knows. She will often tell you that she likes to be alone; but that is not her natural disposition, it is a habit that has grown upon her. Speak to her of nursing, and she is all attention; touch on any other subject, and she has little or no interest. In a few years she becomes *passée*, and perhaps in her nursing career she has had some misfortune, and is not able to employ her time in those little ways so dear to feminine fingers. And yet I do not wish to be gloomy in my view of nurses. They are a good, and, in many instances, a noble and self-denying class of women. But solitude tells upon a nurse, with her constant life among strangers. She loses that happy communion and daily intercourse so dear to us all. The friendship of a few months cannot be called permanent, and she seldom sees her old patients and their relations again. Whatever cleverness and amount of tact she may possess, she is continually going over the same ground. Her life is like the life of a flower, which is plucked for a time, thrown aside, and forgotten.

And now a word to those nurses who are so highly trained in hospitals of the present day. They must remember that many private and district nurses have spent many years in hospitals, yet when they came into private nursing, they had to begin training over again before they could obtain those virtues so necessary to a private nurse, viz., humility, patience, and cheerfulness under all trials. They must remember that the highest trained nurse of ten years ago will be much like what they themselves will be ten years' hence.

And now, one more word to our friend James Macfarlane, if he is still in the hospital (which I rather doubt). He fully deserves all that he has received through The Hospital for his presumption. He must remember that the time has gone by when a woman's only object in life was matrimony. He should have heard the views of a gentleman expressed on the subject a few weeks ago in my hearing. He said that the time had gone by when a man considered it an honour to ask a woman to marry him; now he considers it an honour if she accepts him.

Islands.

[The discussion on James Macfarlane's letter must now close.—Ed. *T. H.*]

### THE IPSWICH NURSES' HOME.

For nearly fourteen years the Nurses' Home at Ipswich, under charge of Miss Agnes Pye, has been doing a useful and varied work. Not only does it supply trained nurses to all who desire and can afford their services, not only does it send nurses to the sick poor; Miss Pye's specialty is the invaluable one of training the neighbours and relations of the poor how to nurse their sick, under the superintendence of a trained visiting nurse. Thus the good work done in the homes of the poor does not terminate with the end of the case, but leaves behind a precious store of knowledge that can be utilised at any time. At present Miss Pye has twenty-five nurses under her management, fifteen of whom are working among the poor of Ipswich and the neighbourhood, while ten are employed for paying cases. So marked has been the appreciation and encouragement the home has met with that it is proposed to increase the staff of nurses. The regard felt throughout the district for the institution is evidenced by the numerous gifts of food, clothing, bed linen, &c., sent there for distribution among such of the poor as need them. People feel sure that their charity will be well bestowed if it goes through the hands of the nurses of the home, who have opportunities the most well-meaning outsider cannot have of distinguishing between real want and ingenious imposture, and who would otherwise often feel disheartened, knowing that the best nursing is of no avail when half the malady is hunger and cold. There can be no doubt that the enlargement of this well-managed and benevolent institution will greatly develop its usefulness.

### A District Matron's Diary.

[Contributions for this column are cordially invited.]

*May 7th.*—One new serious case. Baby Turner, suffering from a complication of diseases, of which bronchitis is the chief. Poultices are applied. Tommy has quite recovered, as I expected. Mrs. Mitchell is getting on splendidly. Poor Grant is not so well; his feet are swelling again. Jones is improving with the weather, as nearly all asthma cases do.

*May 14th.*—One new case. A girl suffering from extreme debility through want of proper food, and worry. We will send nourishment for a time, and try and rouse her to look on life more cheerfully. Poor Baby Turner has just passed away—too many diseases to cure. Grant got so very much worse the doctor advised him to go into the hospital. Mrs. Mitchell is getting about again, and is very grateful for the kindness shown her.

*May 19th.*—One new serious case. Mrs. Armer, suffering from acute inflammation of the bowels. Hot fomentations and poultices are applied; nourishment and medicine given, and the temperature taken twice a day. The young girl we put on the books last week is better for the extra and regular nourishment. Mrs. Mitchell is still our patient; she has got over her confinement well, but has a bad cold, and is very weak. Finding this parish so light just now, I have given nurse charge of a serious case in the next parish, where the work is very heavy.

*May 27th.*—Three new serious cases. Mrs. Ring, a poor old woman I wrote about some time ago, is ill again. I fear this time she will die. She has had several bad attacks of acute bronchitis, is eighty years old, and badly fed. Though she lives quite alone, she scorns the infirmary, and we must do what we can for her. Mrs. Hamer, another old case, poor thing: her last illness was blood poisoning, and now she has inflammation of the lungs. Mrs. Mark, suffering from pleurisy—hot turpentine fomentations are applied. The girl who was suffering from debility is convalescent, and so is Mrs. Mitchell. The chronic cases are much as usual, and nurse still helps in the next parish.

*June 3rd.* One new case.—Mrs. Bell has had a slight fit, and needs care and nourishment. Both Mrs. Ring and Mrs. Hamer have died since my last visit. Poor old Mrs. Ring died alone, as she had lived, for nurse had left her for a short time. Mrs. Hamer nurse did not leave, for she begged nurse to stay with her to the end. Our young girl recovering from debility we are sending to the sea, as the doctor thinks that change of air will bring all things right. The boy Bertie, whom we sent to Margate some weeks ago, is back, and is no better. It is a case of spinal complaint, and I fear he is dying; he has the restlessness of death, and we can do but little for him.

*June 10th.*—Two new cases, the first one of cancer of the breast. I have only seen one case worse, and there is no chance of cure, and very little relief to be got—it is so far advanced. We dress it carefully daily, using plenty of Condy's Fluid to cleanse it, for which she is most thankful. The other is a very sad case of delirium tremens: the wife is a very deserving woman and a careful mother, but the man has been drinking heavily for a month, and lately had a fit. He has to be held down in bed. His "mates" are helping the poor wife as much as they can, and nurse gives her all her spare time. I should like to send him away, but the wife objects. We have lost the little boy suffering from spinal complaint. The other cases are all doing well.

*June 18th.*—Two new serious cases: one a horrible case of ringworm, I doubt whether we shall cure it for some time. Nurse has washed the child and cut all his hair off, and applied ointment ordered by the doctor. The other is a case of diphtheria; the room is kept well aired, carpets are up, and disinfectants used. Hot poultices and gargle are ordered. The boy is through the worst, I believe, but is extremely weak. The man who was suffering from delirium tremens is much quieter, but very ill. His poor wife is in great distress, fearing he will die. The cancer case gets steadily worse.

*June 25th.*—Four new and serious cases. Baby Mitchell is surely dying; he is inwardly convulsed, and has consumption of the bowels. It will be a relief when baby has gone, for the wretched husband has left the wife with four children under four years of age to support. The next is a bad case of pneumonia, her temperature is 104 deg. I trust, though, that we shall save her. Hope, a man suffering from stone and stricture: he is in a dangerous state, and I could only beg him to go alone to the hospital, as he cannot be properly treated at home. Mrs. Best, suffering from hæmorrhage of the lungs; she is not allowed to move, and everything is given her cold, and she always keeps a piece of ice in her mouth. Medicine is given every four hours. I think she will recover, she is so patient and hopeful. The case of diphtheria is doing well. The poor woman with cancer is sinking fast; her pain will soon be over.

### Everybody's Opinion.

[*Correspondence on all subjects is welcome, but correspondents would oblige by writing on one side of the paper only.*]

### NURSING FOR THE MIDDLE CLASSES.

WILL you allow me to inform "Bachelors," and all others who may need nursing for less cost than that supplied by institutions at weekly payments, that wherever a branch of the National and Metropolitan Association for District Nursing is established, nurses who are ladies and have received first-rate training, can be procured for daily visits, one, two, or three, as the case requires, for such payments as can be afforded, the very poor being nursed free. The Central Home is at 23, Bloomsbury Square, and has been at work for the last ten years. There are now several branches at Kensington, Paddington, Holloway, Hampstead, Westminster, Walworth, and Wandsworth, besides others in distant counties. The system of daily visits only is preferred by many to a resident nurse, and I am convinced that the system will ere long be extended to all classes, to their relief and comfort. Not even the most expensive nurse can do both day and night duty.

LOUISA TWINING.

### THE WANT SUPPLIED.

THE discussion which has arisen as to the question of how the middle-class are to obtain skilled nursing, being in many instances unable to pay for it, and ineligible for the help supplied by charity, has interested me greatly, and I venture to think that it may be of interest to those who are stirring in the matter to know that I have, with a friend (who is also a trained nurse), recently started a private nursing institution in one of the suburbs, having the object of meeting this long-felt want specially in view. We have none but thoroughly qualified nurses, and to those who can afford to pay fully for their services we make the usual charges, but we nurse those whose means are insufficient for this for what they can afford to pay; and within reasonable distances we send our nurses by the hour, morning and evening, or for a single night, as they may be required. We hope in time to start a fund to which the rich can contribute to help in the nursing of those requiring what they have not means to pay for. Our institution is, as yet, a small one, and it is with some diffidence that I trespass at so much length on your columns, but I have seen no notice of anything of this kind having been started, and think it may interest some of my fellow-workers to know that some small effort is being made to meet the want.

HELP.

### NURSES FOR NERVOUS AND MENTAL DISORDERS.

THE Institution for Nurses for Nervous and Mental Disorders, 1, Northop Street, Grosvenor Square, is managed on wise and thoughtful principles for the benefit of the nurses, as well as of those who employ them. Probationers, after three months' training in an asylum, or if already trained for mental nursing, after trial in the home, and with patients, are engaged as nurses. They then promise to serve for three years, which engagement they cannot break, except on giving three months' notice to the superintendent, and paying a fine of £6. This engagement is renewable, where the testimonials and conduct have been satisfactory, every three years for a like period. They are allowed an annual stipend of £20, which is raised to £23 after three years' service; £26 after six years' service; and £29 after nine years' service; supplied with an appropriate dress, and maintained in the Home provided for them during the intervals of their cases. At the end of twelve years they have a superannuation pension of £20 a-year when the fund allows. The nurses are engaged from the age of twenty-five; if younger, a deduction is made in their salaries. The Superannuation Fund has been gradually accumulated—first, by payment of 10 per cent. out of the nurses' earnings; secondly, by additional payments out of the balance credit of the current account at Sir S. Scott and Co., 1, Cavendish Square; thirdly, by nurses' fines; and, lastly, by donations from friends. There is also a Gratuity Fund of £60 0*s.* 3*d.* in the Post Office Savings' Bank, for gifts of £5 to nurses after three years' service when invalided, or leaving to be married; £10 after six years' service, with good testimonials and satisfactory conduct; and £15 after nine years' good service. When, however, a nurse accepts a legacy from a patient, she becomes ineligible to participate in the pensions or gratuities of the Institution.

### Notes and Queries.

TO CORRESPONDENTS.—1. Questions may be written on post-cards. 2. Advertisements in disguise are inadmissible. 3. In answering a query please quote the number. 4. If a private answer is desired, a stamped addressed envelope must be forwarded.

(36) *Margareth* wishes to hear of a hospital where she can gain experience as a nurse without paying a premium.—*Margareth* should select the hospital she would prefer, and communicate with the matron.

(35) *M.* will be much obliged by being told in the next number of THE HOSPITAL what is the best thing for washing the face with after using lanoline, which was recommended in THE HOSPITAL a few weeks ago. Soap and water, either hot or cold, does not seem to remove it, but leaves the face sticky and shiny.—Have you tried Pears' soap? ED. *T. H.*

**Answers.**

*Helen Shines* should apply to the Secretary, British Nurses' Association, London.

### The Nurses' Bookshelf.

**Domville's Manual.***

THE sixth edition of this well-known manual has been sent to us, and we are glad nurses have so appreciated its merits as to call for so many editions. It is one of the most concise and thorough works on nursing ever written, and while it can never take the place of fuller teaching, will always be a handy volume to carry about to refresh the memory. The directions for the preparation for post-mortem examination, the chapter on sick-room cookery, and the glossary of medical terms, are all subjects frequently omitted in books on general nursing, though they may have separate volumes to themselves. To carry about Hoblyn's Dictionary is quite unnecessary to the nurse who possesses "Domville's Manual;" such terms as she is likely to meet with in ordinary work are all explained in the glossary in short clear sentences. It is seldom we see a book so devoid of faults, and so much to the point.

### Vacancies.

The following vacancies are announced:—

**Nurses.**

Nurses' Home, Truro; Two.

Ipswich Nurses' Home; £22.

Sheffield Nurses' Home; £25.

Newcastle Infectious Hospital; £25.

Jersey Institute.

Blackheath Nursing Institute.

Hants County Asylum; £17.

Cumberland Infirmary.

Eastern Fever Hospital; £30.

Newport Infirmary; Two.

Swansea Nursing Institute.

Cottage Hospital, Eltham, Kent.

Edinburgh City Poorhouse; £25.

Kingston Nurses' Home, Hull; Several.

Cottage Hospital, Halstead, Essex: Nurse-Matron, £25.

Cottage Hospital, Teddington and Hampton Wick; Nurse Housekeeper, £18 to £20.

Brighton and Hove Lying-in Institution; £30.

Nursing Institution, Ryde, I.W.

**Probationers.**

Stoke-upon-Trent Institute; Two.

**District Nurses.**

Belfast. Stockport.

**Matron.**

Ramsgate Seamen's Infirmary; £40.

### Exchange and Mart.

UNDER this heading we propose to insert purely Exchange advertisements, at a charge of sixpence for eighteen words, or three insertions for one shilling It must be distinctly understood that ordinary advertisements will not be inserted here.

WANTED.—"Quain's Dictionary," in fair condition. Would give a pocket spectroscope, which cost twenty-five shillings. Address Student, Office of THE HOSPITAL.

APRONS.—Half a-dozen white linen aprons with bibs. Will exchange for nurse's surgical case. Nurse Norma, Office of THE HOSPITAL, 38, Old Jewry, E.C.

*A Manual for Hospital Nurses, by Edward J. Domville. (J. and A. Churchill.) Price 2s. 6d.

THE NURSING RECORD.

THE "NURSING RECORD."
A Journal for Nurses and a Chronicle of Hospital and Institution News, &c.
PRICE TWOPENCE. EVERY THURSDAY.

In the interest of a most important profession which is daily increasing in numbers and popularity, it has been found necessary, in order to secure the full and proper representation of the views and claims of those associated with nursing work, to establish the "Nursing Record," a journal which shall in every way faithfully and unreservedly express the opinions and maintain the views of the class it is intended to support.

The Special Objects of the "Nursing Record" are: 1. To deal with matters relating to the nursing profession, more especially those concerning its advancement and consolidation; (2) to protect the general interest of nurses, and to promote their well-being and improvement; (3) to introduce opinion and criticism upon all subjects associated with nursing work; (4) to offer active assistance in the promotion and support of any movement calculated to be of benefit to the nursing profession.

Support and recommendation is earnestly solicited to enable these views to be thoroughly sustained.

SOME PRESS OPINIONS.

"The 'Nursing Record' is a new venture, the first weekly number of which appeared on April 5th. It is described as a journal for nurses, and a chronicle of hospital and institution news. It is, of course, an outcome of the rapidly increasing interest in such works as it relates to, and it seems likely to prove a useful addition to the literature of its class."—*Queen.*

Subscription post free, if paid in advance: Yearly, 8s.; half-yearly, 4s. 6d.; quarterly, 2s. 6d. Specimen copy post free for two stamps.
London: SAMPSON LOW, MARSTON, SEARLE, and RIVINGTON, Limited, St. Dunstan's House, Fetter Lane, Fleet Street, E.C.
**Price Twopence. Every Thursday.**

A JOURNAL FOR NURSES AND A CHRONICLE OF HOSPITAL AND INSTITUTION NEWS, &c.

